# Social distancing between personal belongings during the COVID-19 pandemic

**DOI:** 10.12688/f1000research.130662.2

**Published:** 2024-08-27

**Authors:** Wen Guo, Ayumi Ikeda, Kaito Takashima, Yoshitaka Masuda, Kohei Ueda, Atsunori Ariga, Kyoshiro Sasaki, Yuki Yamada

**Affiliations:** 1 Graduate School of Human-Environment Studies, Kyushu University, Fukuoka, Japan; 2 Japan Society for the Promotion of Science, Tokyo, Japan; 3 Faculty of letters, Chuo University, Hachioji, Japan; 4 Faculty of Informatics, Kansai University, Takatsuki, Japan; 5 Faculty of Arts and Science, Kyushu University, Fukuoka, Japan

**Keywords:** personal space, COVID-19, social distancing, perceived vulnerability to disease (PVD)

## Abstract

**Background:** The COVID-19 pandemic has led to instructions and suggestions from governments and experts to maintain social (physical) distance between people to prevent aerosol transmission of the virus, which is now becoming the norm. Thus, we examined whether the pandemic extended the distance between personal belongings.

**Methods:** We recruited 68 university students and instructed them to place their belongings on a long table following another participant (i.e., confederate). We measured the physical distance between the two belongings (i.e., the participant’s and the confederate’s). We collected data between June 10, 2022 and January 23, 2023. Pre-pandemic data was from Ariga (2016). Analysis was completed with one-tailed *t*-tests.

**Results:** Compared with the pre-pandemic results, via one-tailed *t*-test, the distance between the two belongings during the pandemic was significantly longer. Our results supported the hypothesis that the psychological framework for processing people’s belongings has dramatically changed during this pandemic.

**Conclusion:** This change may have been driven by social distancing practices or an increase in perceived vulnerability to disease. Our results provide new implications for future public spatial design, in other words, not only the distance between people, but also the distance between their belongings.

## Introduction

Social psychology and proxemics have long addressed personal space as the spatial range surrounding the individual ( Sommer, 1959; Hall, 1966). The space around them represents a mediation zone between the body and the environment, contributing to the organization of social life. For example, when this space is encroached by others, we may feel uncomfortable, or may prepare our bodies for defensive responses ( Hall, 1966; Coello and Cartaud, 2021). However, the center of personal space is oneself, but it is not restricted to the physical body; much evidence proposed that the concept of self is expanding in space and timeline. For example, the ‘cutaneous rabbit’ can be felt not only on one’s body but also on an object that one is grasping ( Miyazaki *et al.,* 2010). The line of sight away from the eye can still be perceived as exerting force on the object ( Guterstam *et al.,* 2019). Furthermore, the extension of body representations to objects has been observed in neuroscience ( Iriki *et al.,* 1996). As a result, personal space boundaries also expand with the extension of body perception.

One observed phenomenon is in some school situations where there are desk-mates; after an argument, desk-mates will avoid putting their stationery close to the other’s belongings. It is possible that people develop a tendency to recognize personal belonging as a representation of the self at an early age ( Pedersen, 1973). According to James (1890), the self is the sum of all that he can call his, including all possessions, which gives him the same emotions. Briefly, we regard our possessions as part of ourselves. Is this sense of ownership of objects related to personal space? Ariga (2016) reported that participants placed their own objects at a greater distance from the objects of those who were unfavorable. These findings suggest that individuals’ personal space extended to the space surrounding their belongings (i.e., extended personal space). The placement of such personal belongings can be considered the most significant evidence of an extension of personal space, as it shares the same characteristics with personal space ( Russell & Ward, 1982; Becker, 1973), that the variability depending on the level of familiarity with others.

The COVID-19 pandemic has persisted for more than three years. Governments and experts have provided instructions and suggestions to maintain a certain social (physical) distance between people to prevent droplet and aerosol infection of the virus ( Yonemitsu *et al.,* 2020; Yang *et al.,* 2021), and this is now becoming the norm ( Yamada *et al.,* 2021). Along with the research development, we know that aerosol is one of the transmission routes ( Anderson *et al.,* 2020). Coronaviruses can be released into the surrounding air and exist for an extended period for long-distance transportation ( Jiang *et al.,* 2021), which means that exposed people and objects may carry the coronavirus present in aerosols. Van Doremalen *et al.* (2020) compared the survivability of SARS-CoV-2 (leading to COVID-19) and SARS-CoV-1 on different surfaces. They found that the survival time of SARS-CoV-2 on different material surfaces ranged from a few hours to a few days; suggesting that the surface of an item can be one of the potential pathways for virus transmission. Centers for Disease Control and Prevention (CDC) Guidelines and the World Health Organization (WHO), in their information on COVID-19, also mentioned that the virus can be transmitted by direct contact with contaminated surfaces.

Regardless of the purpose or function of a place, the dense gathering of many people must be avoided. Is it possible to receive such health guidance consistently and imperceptibly influence people’s social cognitive behavior? Recent studies have reported that interpersonal distance (IPD) increased during the COVID-19 pandemic ( Holt *et al.*, 2022; Iachini *et al.*, 2021), and enlarged IPD preferences were predicted to persist beyond the pandemic ( Welsch *et al.,* 2021). The results of an experiment in Arabia revealed that in the post-epidemic era, 76% of the participants were already subjectively reluctant to share close physical distance or socially polite touch with others ( Khan *et al.,* 2021). Responding to precautions, the external manifestation is that we have actively increased our physical distance, but in reality, personal space has also expanded accordingly. In several studies related to personal distance in COVID-19, Fini *et al.* (2021) showed that interpersonal distance is influenced by the perception of realistic threat measured through the COVID-19 threat. In addition, previous research proposed that not only the perception of infection will make the public practice social distancing, but the behavior of practicing social distancing is related to specific individuals as well as related to the social meaning of collective health ( Fazio *et al*., 2021).

How has the COVID-19 pandemic and countermeasures against it changed human-human and human-object interactions? Considering the findings of Ariga (2016), one’s personal space extends to his/her belongings, leaving them with an ‘extended personal space’. The present study aimed to examine if the ‘extended personal space’ increased as well as the personal space that has been affected under the pandemic of COVID-19.

We initially tried to confirm that extended personal space would be larger during the COVID-19 pandemic than during pre-pandemic (i.e., before the pandemic). Maintaining a social (physical) distance from each other is valid for preventing viral infection by droplets and aerosols. Such avoidance of a close distance from other people over a long period modulates personal space ( Tootell *et al.,* 2021; Welsch *et al.,* 2021). Thus, if it is true that space surrounding one’s belongings is based on the interpersonal relationship of their owner ( Ariga, 2016), we predicted that extended personal space would be larger at the present time than at pre-pandemic (i.e., comparing extended personal space in the present time with that in Ariga’s study).

## Methods

### Pre-registration

The present study was pre-registered on the Open Science Framework (OSF) prior to conducting the experiment ( Yamada *et al.,* 2023).

### Ethical statement

Ethical approval for this study was obtained from the Graduate School of Human-Environment Studies, Kyushu University (approval number:2021-030). Written informed consent was obtained from all participants prior to the experiment.

### Participants

The experiment period was from June 10, 2022, to January 23, 2023. The students who participated in the experiment were recruited from the university. We recruited the students via some social networking services (SNS) such as Twitter and LINE. In addition, they contacted us to participate in the experiment by scanning the QR code on our recruitment poster or were directly recruited face-to-face by the experimenters on campus. The inclusion criteria were native Japanese or international students who could speak Japanese proficiently. They were paid 1000 yen to agree to participate. As in Ariga (2016), data were collected from equal numbers of male and female participants in each group. One of the three potential confederates (three of the authors) participated in the experiment in pairs with an external participant.

Power analysis was performed using G*Power software ( Faul *et al.,* 2007). We planned to perform a one-tailed *t*-test on the main hypothesis. There were no related studies; thus, it was difficult to estimate the effect size. In this case, adopting an unbiased effect size (i.e., medium effect size) for power analysis is widely accepted (e.g., Chen and Kao, 2012; Juristo and Moreno, 2003). Considering this and the point when the sample size of Ariga (2016) was *N* = 40, we performed a power analysis (Cohen’s *d* = 0.5, α = .05, 1-β = .80, allocation ratio *N*2/ *N*1 = 1.7), and consequently, the calculated sample sizes were *N* = 40 per group 1 and *N* = 68 per group 2. Therefore, we set *N* = 108 as the required sample size, and hence collected data from 68 participants. The pre-registered exclusion criterion was participants who did not place their own belongings on the table; their data were excluded, and additional participants were recruited until the available participants reached 68. Finally, we collected data from 70 participants; of these, two participants were excluded since they did not bring any belongings. Data from the remaining 68 (male = 34, female = 34, *M* = 20.9, *SD* = 2.2) participants were included in the analysis. All the participants provided written informed consent after a debriefing at the end of the experiment.

### Study design

To examine our main hypothesis, we compared extended personal space before the COVID-19 pandemic (i.e., the pre-pandemic) using Ariga’s (2016) data with that during the pandemic (the present data). There was one between-participants factor in this hypothesis. In addition, to exploratorily investigate whether the data have the same tendency as Ariga (2016), the experiment employed friendly confederate and unfriendly confederate conditions, which was a between-participants factor.

### Procedure

**Figure 1.**
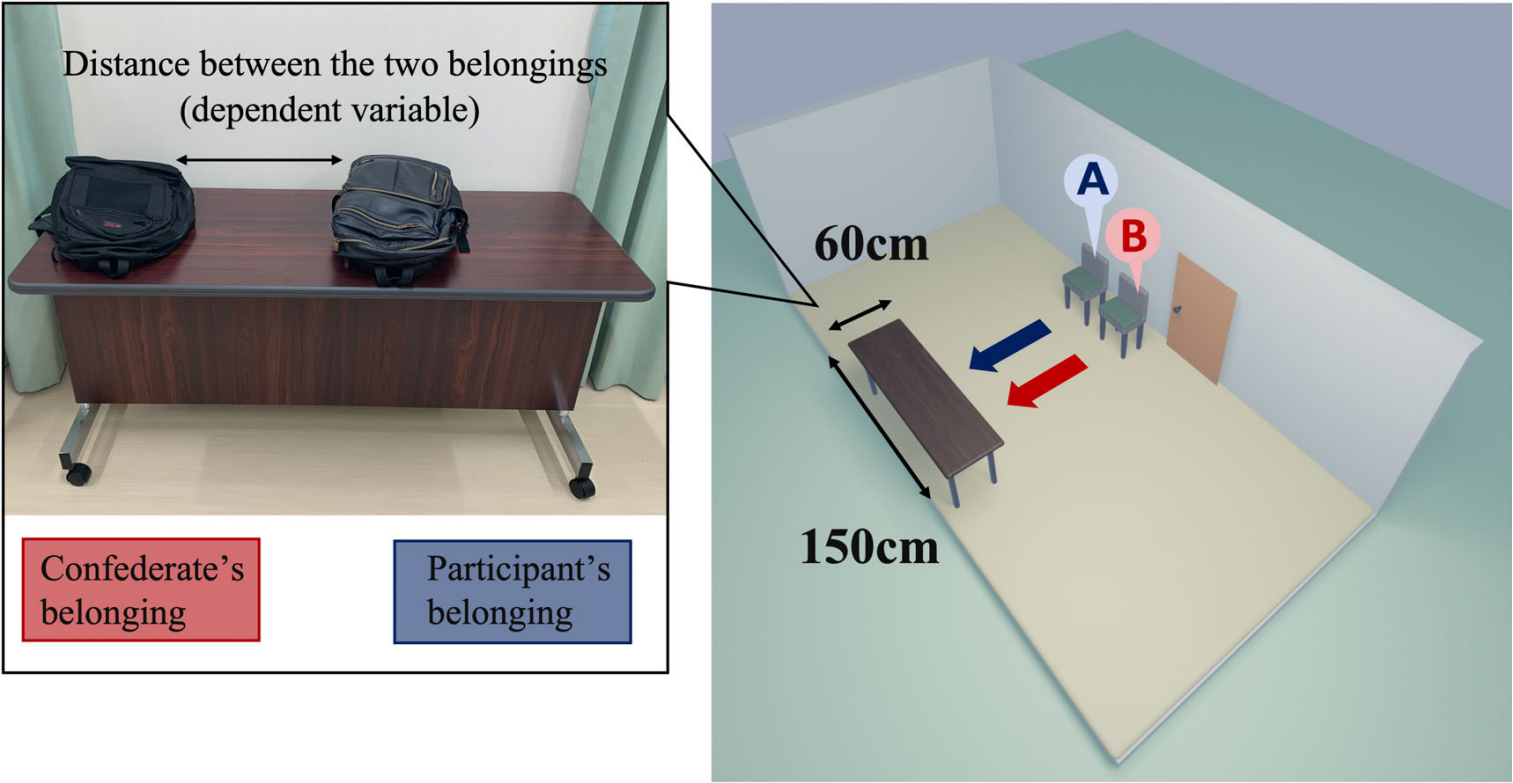
Experimental setting. *Note.* Left panel: A typical positioning of the belongings is depicted. In the experiment, the confederate and participant placed their personal belongings on the table sequentially (first, the confederate placed their backpack on the right side of the table every time). The experimenter measured the minimum distance between the two belongings (accurate to millimeters). Right panel: schematic representation of the experimental room. Two chairs were set up next to the room door: first, the confederate would sit on chair B and then, the participant was asked to sit on chair A, after entering the room. The table (W 60 cm × H 150 cm) for placing their belongings was facing the chairs. The width of the confederate’s belongings was about 36cm. The confederates were three graduate male students who took turns according to their schedules. Their average age was 23.6 years old (the confederate in the original experiment was a 21-years-old male).

The experimental setup is shown in Figure 1. A participant entered the waiting room, where the confederate was already present, and sat next to the confederate’s chair. The distance between two chairs was roughly 30-40 cm. In the friendly condition, the confederate started to talk about the weather with the participant and then tried to continue chatting freely. However, in the unfriendly condition, the confederate ignored the participant’s conversation and sighed every 15 seconds. Then, in both conditions, the experimenter instructed the participant and the confederate to place their belongings on the table and leave the waiting room to the experimental room. The confederate put his belongings in first and then left the room. The experimenter measured the shortest distance between the two belongings and took pictures. Distance was used as the dependent variable: If both belongings were in contact, the distance was coded as 0. In the experimental room, the participants completed a fake task and a rating task of favorability for the other participant (i.e., confederate). After the experiment was completed, we asked the participants two questions: ‘Do you realize our manipulation of favorability for the confederate?’ and ‘Why did you put the belongings there?’

### Analysis plan

We planned to perform the following pre-registered analyses.

*Confirmatory analysis*: First, as our study aims to explore the changes in expanded personal space from pre-pandemic to the pandemic, we compared our results with those of the original study ( Ariga, 2016), using their data. A one-tailed *t*-test was conducted to confirm whether the mean distance between the nearest edges of belongings ( Figure 1, left) would be significantly longer in the present experiment (i.e., the pandemic condition) than in the original (i.e., the pre-pandemic condition). In this and subsequent analyses, the alpha level was set to α = .05 as the inference criterion.

*Exploratory analysis:* For a manipulation check, we conducted a one-tailed *t*-test of the favorability scores for the confederates to confirm whether these scores would be significantly higher in the friendly condition than in the unfriendly condition. Additionally, to test whether there was a difference in the effect of interpersonal relationships on extended personal space between pre-pandemic and the pandemic, a two-way between-participants analysis of variance (ANOVA) with confederate (friendly vs. unfriendly) and experimental timing (pre-pandemic vs. pandemic) as between-participants factors on the mean distance between belongings was conducted. For ANOVA, a significant interaction between these factors would support the hypothesis that there is a difference in the effect of interpersonal relationships on extended personal space between pre-pandemic and the pandemic. Furthermore, the significant main effect of the confederate would indicate that we could have replicated the phenomenon of the original study ( Ariga, 2016).

R 4.1.0 ( R Core Team, 2021) was used for all analyses in this study, and the analysis code is available at OSF ( https://osf.io/sr3x8). Effect size (Cohen's *d*) was calculated by *effectsize* package ( Ben-Shachar, Lüdecke, and Makowski, 2020) version 0.6.0.1. For ANOVA, we used the anovakun function ( Iseki, 2022) version 4.8.7.

## Results

A total of 68 participants, 34 in the unfriendly condition (male = 17, female = 17, *M* = 20.83, *SD* = 2.22) and 34 in the friendly condition (male = 17, female = 17, *M* = 20.97, *SD* = 2.23), participated in the present study.

### Confirmatory analysis

**Figure 2.**
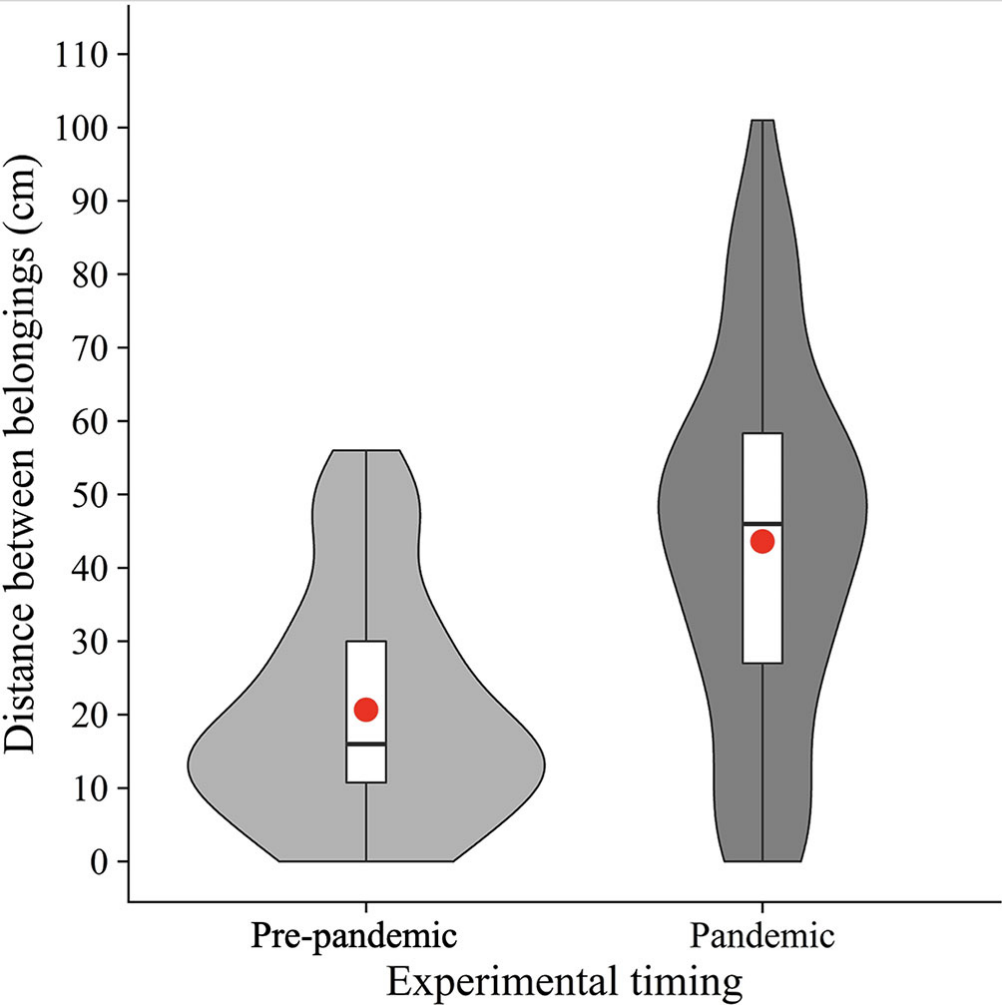
Violin plots and boxplots for the mean distance between belongings (pre-pandemic vs. pandemic). *Note.* Red circles show mean values.

As pre-registered, we calculated the mean distance between the nearest edges of belongings ( Figure 2). Welch’s unpaired *t*-test showed that the mean distance was significantly longer in the pandemic condition ( *M* = 43.6, *SD* = 25.0) than in the pre-pandemic condition ( Ariga, 2016; *M* = 20.6, *SD* = 15.6) ( *t*(105.63) = 5.88, *p* < .001, *d* = 1.10). The results suggest that people place their belongings further away from others in the COVID-19 era, supporting our hypothesis.

### Exploratory analysis

**Figure 3.**
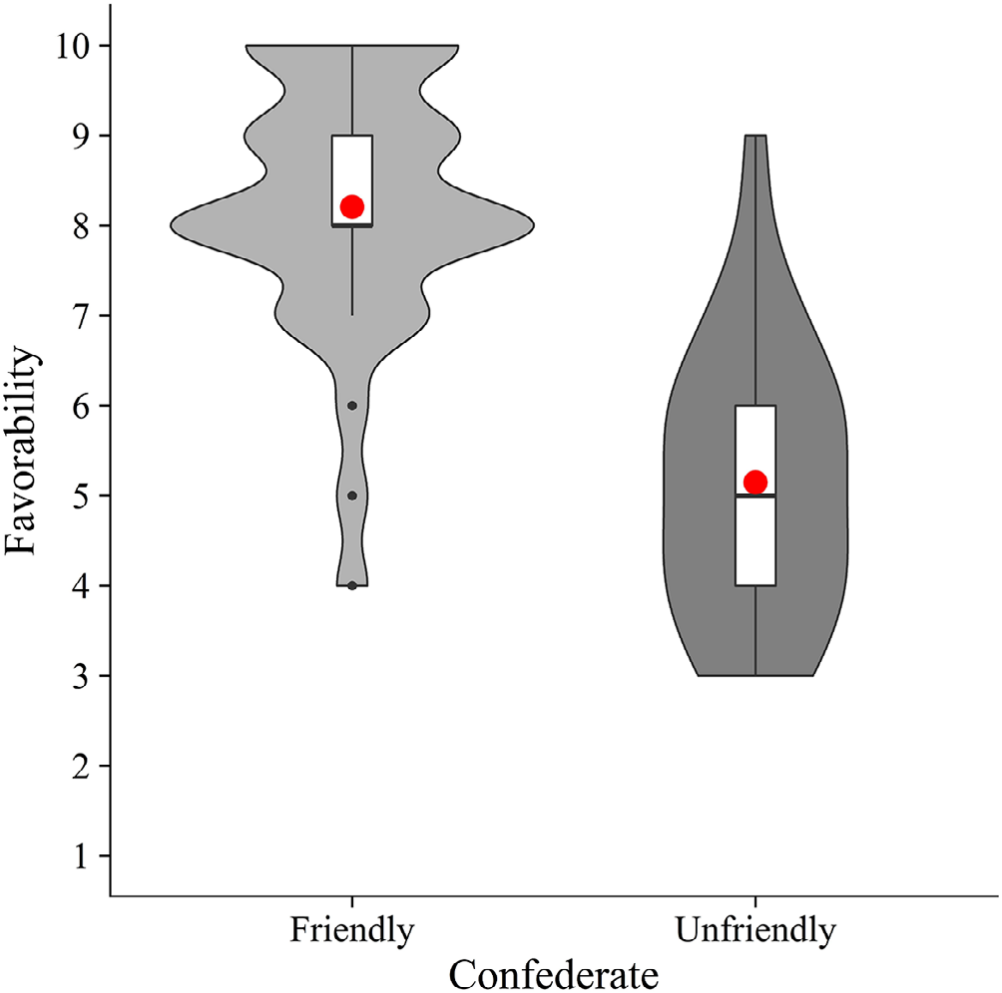
Violin plots and boxplots for the mean favorability of confederate (friendly vs. unfriendly). *Note.* Red circles show mean values.

Next, to check whether our manipulation was successful, we calculated the mean favorability for each condition (friendly and unfriendly) ( Figure 3). Welch’s unpaired *t*-test showed that the friendly condition ( *M* = 8.21, *SD* = 1.43) had a significantly higher mean favorability than the unfriendly condition ( *M* = 5.15, *SD* = 1.52) ( *t*(65.76) = 8.54, *p* < .001, *d* = 2.07). These results suggest that the experimental manipulation was effective.

**Figure 4.**
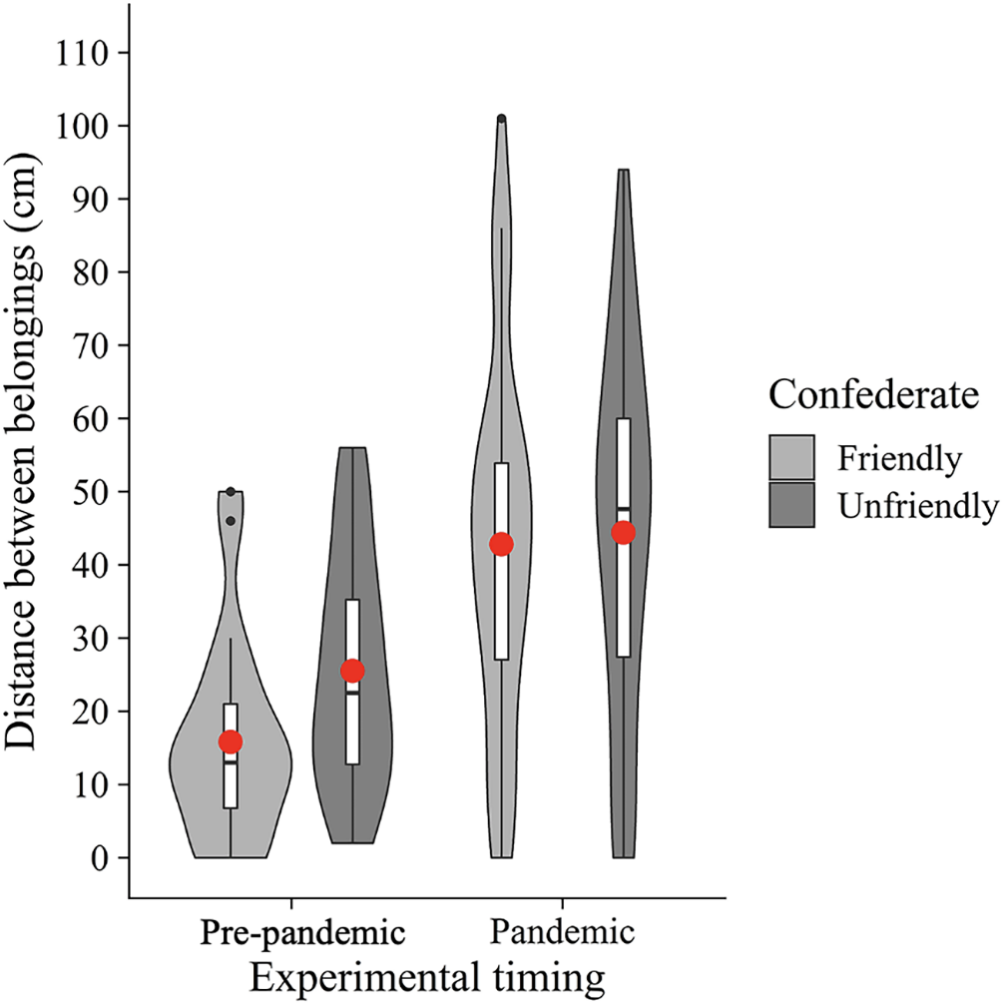
Violin plots and boxplots for the mean distance between belongings (experimental timing × confederate). *Note.* Red circles show mean values.

In addition, we conducted an exploratory two-way between-participants ANOVA with the confederate (friendly vs. unfriendly) and experimental timing (pre-pandemic vs. pandemic) as between-participants factors on the distance between belongings ( Figure 4). The results showed a significant main effect of the experimental timing ( *F*(1, 104) = 27.39, *p* < .001, $\eta_{G}^{2}$ = 0.21). However, neither a significant main effect of the confederate ( *F*(1, 104) = 1.66, *p* = .20, $\eta_{G}^{2}$ = 0.02) nor an interaction ( *F*(1, 104) = 0.85, *p* = .36, $\eta_{G}^{2}$ = 0.01) were found. These results suggest that people place more distance between their belongings and others' in the COVID-19 era, regardless of whether the other participant (i.e., confederate) was friendly.

## Discussion

This study aimed to investigate whether the distance between personal belongings changed during the COVID-19 pandemic. We directly replicated the experiment on the phenomenon in which distance was governed by the interpersonal relationship between owners, called extended personal space ( Ariga, 2016), and explored the effect of COVID-19 on this phenomenon. Compared with the results for pre-pandemic from Ariga (2016), the gross distance between personal belongings during the pandemic was significantly longer. The results suggest that our pre-registered hypothesis was supported; extended personal space increased during period of the COVID-19 pandemic, but we consider COVID-19 as one of the reasons for this change. The large effect size of the difference implies that the psychological framework for processing people’s own belongings changed dramatically during this pandemic.

In this study, the procedure was directly repeated from the original experiment; the participants were Japanese university students, as in Ariga (2016), which ensured that the significant increase in distance was not due to inherent differences in personal space based on ethnicity ( Hall, 1966) or age ( Fry and Willis, 1971). The two experiments differed in period (pre-pandemic vs. pandemic). Participants in this study were university students who were under the COVID-19 pandemic during their university years; they were required to maintain social distance and avoid unnecessary outings. Maintaining social distancing means reducing or minimizing human interactions ( Wilder-Smith and Freedman, 2020). Several studies have confirmed the increase in personal space during the COVID-19 pandemic and suggested the practice of social distancing attributed to the increase ( Fini, Tummolini, and Borghi, 2021; Fazio *et al.,* 2021). Moreover, our study found that the distance between objects increased during the pandemic. Considering Ariga (2016), personal belongings have the same personal space-like properties as the owner, belongings also maintain their social distance when they are placed. This distance could manifest in the elongated distance of personal belongings.

One possibility is that the participants recognized the confederate and their belonging as carriers of an infectious disease. In Japan, perceived vulnerability to disease (PVD) has increased during the COVID-19 pandemic compared to pre-pandemic era ( Yonemitsu *et al.,* 2020), and the perceived risk of COVID-19 infection is associated with increased personal space ( Holt *et al.,* 2022). Since personal belongings also can be the source of infection through aerosols, the perceived risk of infection may extend to them. This suggests that participants without sufficient prior information could place their belongings at a distance from the confederate’s belongings to avoid contamination as they were unsure whether the stranger and their belongings were infected with viruses. Moreover, some previous studies declared that the confined spaces may enhance the feeling of personal space invasion, thereby increasing psychological stress and anxiety ( Aiello *et al*., 1977; Evans, & Wener, 2007). In the present study, for strict replication and fair comparison, the chair distance was set to be the same as in the original experiment conducted in the pre-pandemic period, but the small distance between chairs may have created a sense of social anxiety and constriction at the beginning, making participants place belongings farther away to alleviate seated proximity in subsequent place sessions. No studies that have been done to illustrate the effect of physical proximity on the unraveling of the distance between items.

Another possibility is that participants tried to be compliant. In the US, a 1% increase in new cases (deaths) in the last seven days is associated with a 3% (11%) increase in social distancing intensity ( Besley and Dray, 2022). In Japan, the number of cases and deaths is reported daily in the news which could maintain a high level of compliance (i.e., willingness to distance themselves socially). In fact, all valid participants in this study voluntarily wore face masks, which is a routine infection control measure. We speculated that participants may have maintained a distance between belongings to ensure compliance. This compliance-based explanation can coexist independently with the above explanation of infection avoidance.

Intriguingly, our study did not find a significant effect of the confederate’s friendliness on participants’ behavior, as indicated by the lack of a significant interaction effect in the ANOVA. This result appears to be inconsistent with the findings from Ariga (2016). One possible explanation for this discrepancy could be a ceiling effect, where participants’ behavior might have already reached its peak, limiting the observable impact of additional friendliness. In other words, the width of the desk (150 cm) was long enough in the pre-pandemic period ( Ariga, 2016), but may have been too short to study the effect of the confederate during the pandemic period (see Figure 1). Although the confederate experimenters tried to place their belongings as far as possible to the left corner of the desks, the space left to the participants was estimated to be about 110 cm. The size of 110 cm minus each participant’s belongings was the maximum distance that the participant could set. The distribution of the data in terms of the COVID-19 pandemic reached close to this maximum distance. If the desks were much longer, participants would have been able to place their belongings at a distance from the unfriendly person’s belongings without worrying about the risk of their belongings falling off. The replicability of the original phenomenon in the confederate itself needs to be confirmed by more replication studies.

Although there is a large difference between the present study and Ariga (2016) in terms of the COVID-19 pandemic, other potential differences could have existed as hidden moderators, which might have impacted the results. For example, Rissho University, where the Ariga (2016) experiment was conducted, is in Tokyo, while Kyushu University, where we conducted the present study, is in the deep mountainous countryside. Many of the students at Rissho University, the sample from which the pre-pandemic data was generated, use fully crowded trains to commute to campus every day, possibly affecting one’s personal space.

In addition, about a decade has passed since Ariga’s (2016) data collection and the present study. Therefore, we should consider that changes, unrelated to the pandemic, might have occurred in the long term. For example, in the 2010s, smartphones became widespread in Japan, and hence, social media and social networking services (SNS) have greatly developed ( Ministry of Internal Affairs and Communications, 2017). Although this is just a speculation, the distance for social interaction may have changed over time, as such communication without the effect of physical distance from the other person becomes mainstream. This issue can be addressed in the future by examining the relationship between personal spaces and SNS use. In addition, it would be important to consider other factors such as mood, interpersonal anxiety, and other person-related characteristics.

To dissociate the factor of the pandemic from other potential factors, conducting the study using the same procedure again after the pandemic would be effective. If future research compares the data of Ariga (2016), the present research, and future research, such as a single case design (i.e., ABA design), they can extract the impact of the COVID-19 pandemic to our personal space more clearly than our study. Alternatively, examining the association between the impact and individual differences in disgust proneness ( Haidt, McCauley, and Rozin, 1994; Curtis, De Barra, and Aunger, 2011) or compliance ( Zajenkowski *et al.,* 2020) might be helpful. This pandemic has inspired researchers worldwide, and too many papers were published ( Pai, 2020). However, it is essential to reverify scientific knowledge, including our study, in the post-COVID-19 era. This should lead to strong knowledge in preparation for the next pandemic.

## Conclusion

In summary, this study examined expanded personal space during the COVID-19 pandemic. Personal space is modulated by several factors including gender, personality traits ( Khmiliar *et al.,* 2020), mental status ( Park *et al.,* 2009; Schoretsanitis *et al.,* 2016; Kennedy *et al.*, 2009), and social function (e.g., social cognition; Holt *et al.,* 2015). The distance of personal space serves as a mechanism to avoid disease when the body is threatened by a virus ( Park, 2015). Thus, specific conditions in which the threat of infection is extremely high, such as the COVID-19 pandemic, could motivate people to place belongings at increasing distances from others. Although the present study could not clarify the mechanism of this phenomenon and has several limitations discussed above, the results show that people significantly increased, with a large effect size, the distance between personal belongings during the COVID-19 pandemic. It is important to note, however, that the results of the present experiment do not fully demonstrate that COVID-19 was the only factor that affected the expanded personal space between belongings. Without regard to personal favorability, the distance between personal belongings now is already twice as great as that in pre-pandemic. Our results provide new insights into future spatial design of public spaces. Not only the social distance between people's seats but also personal belongings need their ‘own space’ in open places. For example, but not limited to, lockers (or shelving) in gyms and large public baths should be increased in space to ensure the psychological safety and comfort of users.

## Data Availability

Open Science Framework: The influence of the COVID-19 pandemic on personal space extends to belonging ( https://doi.org/10.17605/OSF.IO/SR3X8) ( Guo *et al., *2023). This project contains the following underlying data:
• Analysis code. (Analysis for *t*-tests and ANOVA of data). • Raw data.csv (Measurements of distance between each participant and confederate’s belongings for friendly and unfriendly settings for current study and Agriga 2016 data). • Picture of the distance between the two belongings.zip. (Pictures of belongings in friendly and unfriendly settings.) Data are available under the terms of the Creative Commons Attribution 4.0 International license (CC-BY 4.0).
