# Supplementary material for: Social distancing between personal belongings during the COVID-19 pandemic
Source: F1000Res. 2025 Feb 5;12:199. Originally published 2023 Feb 20. [Version 3] doi: 10.12688/f1000research.130662.3 (PMC11822249; doi:10.12688/f1000research.130662.3)
Supplement: Peer review history

# Reviewer report: Reviewer response for version 3

**DOI:** 10.5256/f1000research.177402.r364741

**Published:** 2025-02-17

**Authors:** Tomoko Isomura

**Affiliations:** 1 Graduate School of Informatics, Nagoya University, Nagoya, Japan

I sincerely apologize for my delayed response due to issues with receiving notifications.

 I appreciate the authors' efforts in addressing my concerns.

 One thing I am still wondering about is their raw data. While the authors mentioned that there are 68 data after excluding two participants who did not bring any belongings, I found that the raw data includes all 70 participants with "distance" data.

 What do the distance data from these two participants represent then?

 I have no further concerns beyond this.

Is the work clearly and accurately presented and does it cite the current literature?

Yes

If applicable, is the statistical analysis and its interpretation appropriate?

Partly

Are all the source data underlying the results available to ensure full reproducibility?

Partly

Is the study design appropriate and is the work technically sound?

Partly

Are the conclusions drawn adequately supported by the results?

Partly

Are sufficient details of methods and analysis provided to allow replication by others?

Partly

Reviewer Expertise:

experimental psychology; embodied cognition; social cognition

I confirm that I have read this submission and believe that I have an appropriate level of expertise to confirm that it is of an acceptable scientific standard, however I have significant reservations, as outlined above.

# Reviewer report: Reviewer response for version 3

**DOI:** 10.5256/f1000research.177402.r365484

**Published:** 2025-02-17

**Authors:** Genta Miyazaki

**Affiliations:** 1 Gakushuin University, Tokyo, Japan

**Summary:**

 This pre-registered study examined whether individuals placed their personal belongings at a greater distance from others’ belongings during the COVID-19 pandemic than before. The authors compared experimental results from the COVID-19 pandemic with those from the pre-pandemic (Ariga, 2016). They found that personal belongings were spaced farther during the pandemic than before. The authors interpreted these findings as suggesting that as personal space expanded during the COVID-19 pandemic, the extended personal space, represented by the distance between belongings, also increased.

**Review:**

 This study is valuable because it carefully replicates the experimental procedures of the previous research (Ariga, 2016 [Ref-1]). Furthermore, it provides crucial data on extended personal spaces during the COVID-19 pandemic.

 However, as the authors acknowledged in their discussion, the present study did not provide definitive evidence that the observed differences were caused directly by the COVID-19 pandemic. It may be helpful to analyze the responses to the question, "Why did you put the belongings there?" as measured in the present study, to address this limitation. How many participants indicated concerns about COVID-19 or adherence to social distancing?

 This analysis could help reinforce the interpretation of the authors’ findings. In a previous study (Ariga, 2016 [Ref-1]), responses to the same question were analyzed. This revealed that among the 20 participants in the unfriendly condition, 16 stated that they placed their bags away from the confederate’s bag because they did not want to put their belongings close to it. This suggests that the participants could verbalize their reasons for maintaining distance between their belongings and those of the confederate.

**Minor Points:**

1. The variance in the distance between belongings appeared to be greater during the pandemic than during the pre-pandemic period. Please provide an interpretation of this result.
2. The manuscript states, "Intriguingly, our study did not find a significant effect of the confederate’s friendliness on participants’ behavior, as indicated by the lack of a significant interaction effect in the ANOVA" (p. 9). However, if the present experiment observed an effect of the confederate’s friendliness similar to that in Ariga (2016 [Ref-1]), an interaction should not have been expected. Instead of stating "the lack of a significant interaction effect," the sentence should refer to "the lack of a significant main effect of the confederate’s friendliness".

Is the work clearly and accurately presented and does it cite the current literature?

Yes

If applicable, is the statistical analysis and its interpretation appropriate?

Yes

Are all the source data underlying the results available to ensure full reproducibility?

Yes

Is the study design appropriate and is the work technically sound?

Partly

Are the conclusions drawn adequately supported by the results?

Partly

Are sufficient details of methods and analysis provided to allow replication by others?

Yes

Reviewer Expertise:

social psychology; interpersonal relationships

I confirm that I have read this submission and believe that I have an appropriate level of expertise to confirm that it is of an acceptable scientific standard, however I have significant reservations, as outlined above.

## References

[1] : [Expansive personal space: Distance between personal belongings reflects the interpersonal distance of their owners]. *Shinrigaku Kenkyu* .2016;87(2) : 10.4992/jjpsy.87.15306 186-90 10.4992/jjpsy.87.15306 27476269

# Reviewer report: Reviewer response for version 3

**DOI:** 10.5256/f1000research.177402.r364740

**Published:** 2025-02-12

**Authors:** Nur Givon-Benjio

**Affiliations:** 1 University of Haifa, Haifa, Israel

The authors have thoroughly addressed all of my concerns.

Is the work clearly and accurately presented and does it cite the current literature?

Partly

If applicable, is the statistical analysis and its interpretation appropriate?

Yes

Are all the source data underlying the results available to ensure full reproducibility?

No source data required

Is the study design appropriate and is the work technically sound?

Yes

Are the conclusions drawn adequately supported by the results?

Partly

Are sufficient details of methods and analysis provided to allow replication by others?

Partly

Reviewer Expertise:

Interpersonal distance (preference, perception) in COVID-19 as well as in clinical populations (social anxiety, autism).

I confirm that I have read this submission and believe that I have an appropriate level of expertise to confirm that it is of an acceptable scientific standard.

# Reviewer report: Reviewer response for version 3

**DOI:** 10.5256/f1000research.177402.r364739

**Published:** 2025-02-12

**Authors:** Leon O. H Kroczek

**Affiliations:** 1 University of Regensburg, Regensburg, Germany

The authors have responded to all of my comments. This includes more details with respect to the experimental setting and a more nuanced discussion of the results with respect to other factors which might have driven the effect.

Is the work clearly and accurately presented and does it cite the current literature?

Partly

If applicable, is the statistical analysis and its interpretation appropriate?

Yes

Are all the source data underlying the results available to ensure full reproducibility?

Yes

Is the study design appropriate and is the work technically sound?

No

Are the conclusions drawn adequately supported by the results?

No

Are sufficient details of methods and analysis provided to allow replication by others?

Partly

Reviewer Expertise:

interpersonal distance, socio-affective behavior

I confirm that I have read this submission and believe that I have an appropriate level of expertise to confirm that it is of an acceptable scientific standard.

# Reviewer report: Reviewer response for version 2

**DOI:** 10.5256/f1000research.170298.r348889

**Published:** 2024-12-31

**Authors:** Nur Givon-Benjio

**Affiliations:** 1 University of Haifa, Haifa, Israel

The current study examined changes in the distance at which people placed their belongings before and after the COVID-19 outbreak in Japan. The authors employed an ecological task simulating a real-life scenario. The results suggest that the pandemic predicted an enlargement (or expansion) of the distance people placed their belongings from those of a confederate. This effect was not explained by whether the confederate was friendly or hostile, nor by a time*confederate interaction.

 Overall, I found the study very interesting and with the potential to make a meaningful contribution to existing knowledge. However, there are several areas that warrant further clarification and consideration:

 1. A clear distinction should be made between interpersonal distance, which refers to the space between people (as discussed by Hall), and peripersonal distance, the space immediately surrounding the body within reach (as explored by Coello and Cartaud). This differentiation is critical for framing the study's context. The fear of infection during the COVID-19 pandemic is primarily linked to interpersonal distance. Studies have shown that fear of infection correlates with a preference for greater interpersonal distance and an underestimation of interpersonal space. However, these effects are tied to subjective perceptions of risk rather than objective risk of infection (Givon-Benjio et al., 2024). On the other hand, personal belongings conceptually align more with peripersonal distance. For example, Lenglart et al. (2023) demonstrated that personal items are perceived as more "within reach" compared to other objects, indicating an expansion of peripersonal distance representation.

 The paper by Coello and Cartaud (2021), cited in the introduction, suggests that interpersonal and peripersonal distances are interlinked and together influence social behavior. I recommend moving this discussion to the end of the paragraph, after clarifying the distinction between the two concepts. Additionally, the innovative nature of this study should be explicitly mentioned—specifically, its combination of these two concepts, typically studied independently, to provide novel insights into how they are interconnected.

 This distinction and integration should also be revisited in the discussion section. Existing literature suggests that the perception of distance is highly context-dependent. For example, would the effect still emerge when the perceived risk is low? Would it manifest if participants were asked to place items they do not own? Or, on the other hand, could the effect persist, as suggested by the lack of influence of the confederate manipulation on the results? Addressing these contextual factors could be done as suggestions for future studies, as answering them may lead to different theoretical and practical conclusions.

 Lenglart, L., Cartaud, A., Quesque, F., Sampaio, A., & Coello, Y. (2023 [Ref - 1]). Object coding in peripersonal space depends on object ownership. Quarterly Journal of Experimental Psychology, 76(8), 1925-1939.

 Givon-Benjio, N., Sokolover, H., Aderka, I. M., Hadad, B. S., & Okon-Singer, H. (2024 [Ref -2]). Perception of interpersonal distance and social distancing before and during COVID-19 pandemic. Scientific Reports, 14(1), 4568.

 2. If the authors use the term "personal space" to refer specifically to peripersonal distance, they should explicitly adopt this terminology throughout the manuscript and to update the literature review, especially after the first paragraph. Alternatively, if "personal space" is intended to mean something different from the space surrounding the body, they should clearly define the term.

 3. The discussion on interpersonal distance (IPD) during COVID-19 in the introduction is incomplete. While the authors cite studies demonstrating an enlargement of IPD following the pandemic, the paragraph overlooks important nuances. For instance, Iachini et al. (2021), which is already cited, found that the increase in IPD occurred only when subjective risk was high. Similarly, Givon-Benjio et al. (2024) compared data from before and after the outbreak and observed that subjective risk, rather than actual risk (e.g., infection rate), predicted changes in preferred interpersonal distance. Even when the actual risk was high, preferred interpersonal distance remained unchanged. These findings are further supported Cartaud et al., 2020).

 The introduction should reflect that the enlargement of IPD is not a universal or conclusive effect but is highly dependent on subjective risk levels. While the authors touch on subjective risk in the discussion, particularly when discussing perceived vulnerability to disease (PVD), this concept should receive greater emphasis both in the introduction and discussion to provide a more nuanced understanding of the findings.

 Cartaud, A., Quesque, F. & Coello, Y. Wearing a face mask against Covid-19 results in a reduction of social distancing. Plos One 15(12), e0243023 (2020 [Ref-3]).

 4. Supporting the findings is a study that found that COVID-19 is linked to reduction in peripersonal distance, which can help explain why people want to put their belonging further away.

 Serino, S. et al. Sharpening of peripersonal space during the COVID-19 pandemic. Curr. Biol. 31(14), R889–R890 (2021 [Ref - 4]).

 5. The fact that participants wore face masks should be mentioned earlier in the text, specifically in the Methods section, as it is a relevant procedural detail. Additionally, this should be discussed further in the Discussion section, with reference to studies examining the relationship between mask-wearing and interpersonal or peripersonal distance.

 Minor:

 6.I found the observations about the role of smartphones and their potential impact on personal space particularly insightful in the discussion.

 7. I suggest reconsidering the inclusion of Figure 3, as it primarily displays the results of a manipulation check, and may not add value to the main narrative of the paper.

Is the work clearly and accurately presented and does it cite the current literature?

Partly

If applicable, is the statistical analysis and its interpretation appropriate?

Yes

Are all the source data underlying the results available to ensure full reproducibility?

No source data required

Is the study design appropriate and is the work technically sound?

Yes

Are the conclusions drawn adequately supported by the results?

Partly

Are sufficient details of methods and analysis provided to allow replication by others?

Partly

Reviewer Expertise:

Interpersonal distance (preference, perception) in COVID-19 as well as in clinical populations (social anxiety, autism).

I confirm that I have read this submission and believe that I have an appropriate level of expertise to confirm that it is of an acceptable scientific standard, however I have significant reservations, as outlined above.

## References

[1] : Object coding in peripersonal space depends on object ownership. *Q J Exp Psychol (Hove)* .2023;76(8) : 10.1177/17470218221128306 1925-1939 10.1177/17470218221128306 36113191

[2] : Perception of interpersonal distance and social distancing before and during COVID-19 pandemic. *Sci Rep* .2024;14(1) : 10.1038/s41598-024-55218-y 4568 10.1038/s41598-024-55218-y 38403693 PMC10894866

[3] : Wearing a face mask against Covid-19 results in a reduction of social distancing. *PLoS One* .2020;15(12) : 10.1371/journal.pone.0243023 e0243023 10.1371/journal.pone.0243023 33284812 PMC7721169

[4] : Sharpening of peripersonal space during the COVID-19 pandemic. *Curr Biol* .2021;31(14) : 10.1016/j.cub.2021.06.001 R889-R890 10.1016/j.cub.2021.06.001 34314711 PMC8188303

# Response

**Published:** 2025-01-22

**Authors:** Kaito Takashima

**Affiliations:** Kyushu University, Japan

The current study examined changes in the distance at which people placed their belongings before and after the COVID-19 outbreak in Japan. The authors employed an ecological task simulating a real-life scenario. The results suggest that the pandemic predicted an enlargement (or expansion) of the distance people placed their belongings from those of a confederate. This effect was not explained by whether the confederate was friendly or hostile, nor by a time*confederate interaction.

 Overall, I found the study very interesting and with the potential to make a meaningful contribution to existing knowledge. However, there are several areas that warrant further clarification and consideration:

 1.     A clear distinction should be made between interpersonal distance, which refers to the space between people (as discussed by Hall), and peripersonal distance, the space immediately surrounding the body within reach (as explored by Coello and Cartaud). This differentiation is critical for framing the study's context. The fear of infection during the COVID-19 pandemic is primarily linked to interpersonal distance. Studies have shown that fear of infection correlates with a preference for greater interpersonal distance and an underestimation of interpersonal space. However, these effects are tied to subjective perceptions of risk rather than objective risk of infection (Givon-Benjio et al., 2024). On the other hand, personal belongings conceptually align more with peripersonal distance. For example, Lenglart et al. (2023) demonstrated that personal items are perceived as more "within reach" compared to other objects, indicating an expansion of peripersonal distance representation.

 The paper by Coello and Cartaud (2021), cited in the introduction, suggests that interpersonal and peripersonal distances are interlinked and together influence social behavior. I recommend moving this discussion to the end of the paragraph, after clarifying the distinction between the two concepts. Additionally, the innovative nature of this study should be explicitly mentioned—specifically, its combination of these two concepts, typically studied independently, to provide novel insights into how they are interconnected.

 This distinction and integration should also be revisited in the discussion section. Existing literature suggests that the perception of distance is highly context-dependent. For example, would the effect still emerge when the perceived risk is low? Would it manifest if participants were asked to place items they do not own? Or, on the other hand, could the effect persist, as suggested by the lack of influence of the confederate manipulation on the results? Addressing these contextual factors could be done as suggestions for future studies, as answering them may lead to different theoretical and practical conclusions.

 Lenglart, L., Cartaud, A., Quesque, F., Sampaio, A., & Coello, Y. (2023 [Ref - 1]). Object coding in peripersonal space depends on object ownership. Quarterly Journal of Experimental Psychology, 76(8), 1925-1939.

 Givon-Benjio, N., Sokolover, H., Aderka, I. M., Hadad, B. S., & Okon-Singer, H. (2024 [Ref -2]). Perception of interpersonal distance and social distancing before and during COVID-19 pandemic. Scientific Reports, 14(1), 4568.

**Response:**

 Thank you for acknowledging the innovative aspects of our study, and highlighting the importance of clearly distinguishing between interpersonal distance (IPD) and peripersonal distance (PPD). Following your suggestion, we have made the following revisions. Firstly, in the first paragraph of introduction, we clearly define and describe interpersonal space and peripersonal space. Then, at the end of the introduction, the innovation point of this paper is added, that is, it combines these two concepts for the first time and discusses their influence on social behavior. Finally,we expanded the discussion on future research direction by incorporating new questions based on your suggestions to highlight how contextual factors may influence our findings.These additions provide a stronger foundation for future studies investigating how interpersonal and peripersonal space interact under different conditions.

 We sincerely appreciate your constructive feedback, as it has significantly enhanced the theoretical clarity and coherence of our manuscript. 

**Revised version: use underline__**

 …Social psychology and proxemics have long addressed personal space as the spatial range surrounding the individual (Sommer, 1959; Hall, 1966). **The space around the individual may exist in two forms. One is interpersonal distance, the area around the body that individuals maintain between themselves and others during social interactions (Candini et al., 2021). The other one is peripersonal space, which is an adaptive and flexible interface between the body and the environment ** The space around them represents a mediation zone between the body and the environment, contributing to the organization of social life. For example, when this space is encroached by others, we may feel uncomfortable or may prepare our bodies for defensive responses (Hall, 1966; Coello and Cartaud, 2021). ………

 …How has the COVID-19 pandemic and countermeasures against it changed human-human and human-object interactions? Considering the findings of Ariga (2016), one’s personal space extends to his/her belongings, leaving them with an ‘extended personal space’. The present study aimed to examine if the ‘extended personal space’ increased as well as the personal space that has been affected under the pandemic of COVID-19. **The fear of infection during the COVID-19 pandemic is closely associated with interpersonal distance. Research indicates that this fear is linked to a tendency to prefer greater interpersonal distance and to underestimate interpersonal space. However, these tendencies stem from subjective perceptions of risk rather than an actual objective risk of infection (Givon-Benjio et al., 2024). On the other hand, personal belongings are more closely related to peripersonal distance. For instance, Lenglart et al. (2023) found that individuals perceive personal items as being more "within reach" compared to other objects, suggesting an expansion in the representation of peripersonal distance. Based on the Coello and Cartaud, they indicated that interpersonal and peripersonal distances are interlinked and together influence social behavior. Therefore, in this study, we innovatively combine these two concepts for the first time to explore the impact of the combination of these two forms of personal space on social actions.**

**Discussion:**

 To dissociate the factor of the pandemic from other potential factors, conducting the study using the same procedure again after the pandemic would be effective. **Additionally, future studies should further examine how interpersonal and peripersonal distances interact in different contexts. For example, the level of perceived infectability (i.e., beliefs about one’s own susceptibility to infectious diseases) in 2023 has dropped to the same level as before the COVID-19 pandemic in Japan (Ikeda et al., 2024). Although the number of diagnosed COVID-19 cases remains high, people's subjective risk perception has declined. The potential changes in sensitivity suggested by such reports provide us with numerous possibilities for discussion. Would the effect still emerge when the perceived risk of infection is low? Additionally, would it manifest if participants were asked to place items they do not own? Alternatively, the persistence of the effect—despite the lack of influence of the confederate’s friendliness—suggests that the expansion of peripersonal space during the pandemic might be independent of social cues. Furthermore, the perception of distance may be shaped by long-term societal and technological changes. The widespread use of smartphones and social media in the 2010s might have altered norms around physical proximity and social interaction. Future research could investigate whether personal space norms adapt to such technological shifts and how these changes interact with interpersonal and peripersonal distances.**

**Added references:**

 Candini, M., Battaglia, S., Benassi, M., di Pellegrino, G., & Frassinetti, F. (2021). The physiological correlates of interpersonal space. *Scientific Reports*, *11*(1), 2611. https://doi.org/10.1038/s41598-021-82223-2

 Lenglart, L., Cartaud, A., Quesque, F., Sampaio, A., & Coello, Y. (2023). Object coding in peripersonal space depends on object ownership. *Quarterly Journal of Experimental Psychology*, *76*(8), 1925-1939. https://doi.org/10.1177/17470218221128306

 Givon-Benjio, N., Sokolover, H., Aderka, I. M., Hadad, B. S., & Okon-Singer, H. (2024). Perception of interpersonal distance and social distancing before and during COVID-19 pandemic. *Scientific Reports*, *14*(1), 4568. https://doi.org/10.1038/s41598-024-55218-y

Ikeda A, Sasaki K, Yamada Y.: Evolution of perceived vulnerability to infection in Japan during the COVID-19 pandemic. Qeios. 2024. https://doi.org/10.32388/4CO88Y.4

 2. If the authors use the term "personal space" to refer specifically to peripersonal distance, they should explicitly adopt this terminology throughout the manuscript and to update the literature review, especially after the first paragraph. Alternatively, if "personal space" is intended to mean something different from the space surrounding the body, they should clearly define the term.

**Response：**

 Thank you for confirming the uniformity of terms. In the present study, "personal space" refers to the surrounding space centered on one's body that is not invaded by others in social activities. Closer to the concept of peripersonal space. In addition, the personal space extended by personal belongings is also closer to the "peripersonal space". We clarified the definition of peripersonal space in the first paragraph of introduction and the reference to personal space in our manuscript to ensure the clear and consistent use of terms.

**Revised version:**

 Social psychology and proxemics have long addressed personal space as the spatial range surrounding the individual ( Sommer, 1959; Hall, 1966). **The space around the individual may exist in two forms. One is interpersonal distance, the area around the body that individuals maintain between themselves and others during social interactions (Candini et al., 2021). The other one is peripersonal space, which is an adaptive and flexible interface between the body and the environment, ** contributing to the organization of social life. For example, when this space is encroached by others, we may feel uncomfortable or may prepare our bodies for defensive responses ( Hall, 1966; Coello and Cartaud, 2021) . **In our study, the concept of 'personal space,' as referenced later, aligns more closely with the notion of peripersonal space. This refers to the space immediately surrounding the body, within reach, centered on the individual, and typically protected from intrusion during social interactions.**

 One observed phenomenon is in some school situations where there are desk-mates; after an argument, desk-mates will avoid putting their stationery close to the other’s belongings. It is possible that people develop a tendency to recognize personal belonging as a representation of the self at an early age ( Pedersen, 1973). According to James (1890), the self is the sum of all that he can call his, including all possessions, which gives him the same emotions. Briefly, we regard our possessions as part of ourselves. **This intrinsic association between personal belongings and the self-forms the foundation for understanding how external objects, including tools and technologies, can become integrated into one’s sense of self, extending the boundaries of personal space. Studies on body ownership illusions suggest that the integration of external objects or virtual representations into one’s sense of self occurs through multisensory signals, highlighting the neural mechanisms underlying extended personal space (Nilsson & Kalckert, 2021).  ** Is this sense of ownership of objects related to personal space? Ariga (2016) reported that participants placed their own objects at a greater distance from the objects of those who were unfavorable. These findings suggest that individuals’ personal space extended to the space surrounding their belongings (i.e., extended personal space). The placement of such personal belongings can be considered the most significant evidence of an extension of personal space, as it shares the same characteristics with personal space ( Russell & Ward, 1982; Becker, 1973), that the variability depending on the level of familiarity with others. **Moreover, recent studies have shown that object ownership influences the representation of peripersonal space. Specifically, self-owned objects within the peripersonal space are processed faster and perceived as more reachable compared to objects owned by others, suggesting an extension of peripersonal space representation around self belongings (Lenglart et al., 2023). This effect is particularly pronounced in individuals with higher interpersonal sensitivity, as their peripersonal space boundaries dynamically adapt in response to social contexts. Further supporting this perspective, Rabellino et al. (2020) highlight that peripersonal space is not only critical for physical interactions with the environment but also closely tied to bodily self-consciousness. They suggest that  peripersonal space representation involves multisensory processing and serves both protective and social functions, allowing individuals to dynamically adjust to interactions with others. These findings further support the idea that ownership can extend personal space to include the area surrounding one's possessions, reinforcing the concept of "extended personal space."**

**Added references**

Lenglart, L., Cartaud, A., Quesque, F., Sampaio, A., & Coello, Y. (2023). Object coding in peripersonal space depends on object ownership. *Quarterly Journal of Experimental Psychology*, *76*(8), 1925-1939. https://doi.org/10.1177/17470218221128306

Nilsson, M., & Kalckert, A. (2021). Region‐of‐interest analysis approaches in neuroimaging studies of body ownership: An activation likelihood estimation meta‐analysis. *European Journal of Neuroscience*, *54*(11), 7974-7988. https://doi.org/10.1111/ejn.15534

Rabellino, D., Frewen, P. A., McKinnon, M. C., & Lanius, R. A. (2020). Peripersonal space and bodily self-consciousness: implications for psychological trauma-related disorders. *Frontiers in Neuroscience*, *14*, 586605. https://doi.org/10.3389/fnins.2020.586605

 3. The discussion on interpersonal distance (IPD) during COVID-19 in the introduction is incomplete. While the authors cite studies demonstrating an enlargement of IPD following the pandemic, the paragraph overlooks important nuances. For instance, Iachini et al. (2021), which is already cited, found that the increase in IPD occurred only when subjective risk was high. Similarly, Givon-Benjio et al. (2024) compared data from before and after the outbreak and observed that subjective risk, rather than actual risk (e.g., infection rate), predicted changes in preferred interpersonal distance. Even when the actual risk was high, preferred interpersonal distance remained unchanged. These findings are further supported Cartaud et al., 2020).

 The introduction should reflect that the enlargement of IPD is not a universal or conclusive effect but is highly dependent on subjective risk levels. While the authors touch on subjective risk in the discussion, particularly when discussing perceived vulnerability to disease (PVD), this concept should receive greater emphasis both in the introduction and discussion to provide a more nuanced understanding of the findings.

 Cartaud, A., Quesque, F. & Coello, Y. Wearing a face mask against Covid-19 results in a reduction of social distancing. Plos One 15(12), e0243023 (2020 [Ref-3]).

**Response:**

 We appreciate your insightful comments on the nuanced relationship between interpersonal distance and subjective risk during the COVID-19 pandemic.

 In response, we have revised the introduction to provide a more balanced discussion on the enlargement of interpersonal distance, emphasizing that this phenomenon is highly dependent on subjective risk levels rather than being a universal or conclusive effect. Specifically, we have incorporated findings from Iachini et al. (2021), Givon-Benjio et al. (2024), and Cartaud et al. (2020) to illustrate how subjective risk plays a crucial role in modulating interpersonal distance. Furthermore, we have strengthened the connection between subjective risk and the concept of extended personal space in the discussion part, which forms the core focus of our study. For details revise, please see "Revised version"

**Revised version:**

 …Recent studies have reported that interpersonal distance (IPD) increased during the COVID-19 pandemic ( Holt *et al.*, 2022; Iachini *et al.*, 2021), and enlarged IPD preferences were predicted to persist beyond the pandemic ( Welsch *et al.,*2021). **However, it is important to note that the enlargement of IPD is not a universal phenomenon. As Iachini et al. (2021) observed, IPD increases only when subjective risk levels are high. Similarly, Givon-Benjio et al. (2024) demonstrated that subjective risk, rather than actual infection risk, predicted changes in preferred IPD. Cartaud et al. (2020) further supported this by showing that even under conditions of high actual risk, individuals’ preferred IPD remained unchanged unless subjective risk was elevated. ** The results of an experiment in Arabia revealed that in the post-epidemic era, 76% of the participants were already subjectively reluctant to share close physical distance or socially polite touch with others ( Khan *et al.,* 2021). Responding to precautions, the external manifestation is that we have actively increased our physical distance, but in reality, personal space has also expanded accordingly. In several studies related to personal distance in COVID-19, Fini *et al.* (2021)showed that interpersonal distance is influenced by the perception of realistic threat measured through the COVID-19 threat. In addition, previous research proposed that not only the perception of infection will make the public practice social distancing, but the behavior of practicing social distancing is related to specific individuals as well as related to the social meaning of collective health ( Fazio *et al*., 2021).

 Discussion :

 Since personal belongings also can be the source of infection through aerosols, the perceived risk of infection may extend to them. **It is precisely due to the increased subjective perception of risk that, in the context of COVID-19, the extension of personal belongings comes to represent the characteristics of personal space, and the distance between personal belongings also increases. Participants** without sufficient prior information could place their belongings at a distance from the confederate’s belongings to avoid contamination as they were unsure whether the stranger and their belongings were infected with viruses.

 4. Supporting the findings is a study that found that COVID-19 is linked to reduction in peripersonal distance, which can help explain why people want to put their belonging further away.

 Serino, S. et al. Sharpening of peripersonal space during the COVID-19 pandemic. Curr. Biol. 31(14), R889–R890 (2021 [Ref - 4]).

**Response:**

  Thank you for pointing out this relevant study by Serino et al. (2021). We agree that their findings on the sharpening of peripersonal space during the COVID-19 pandemic provide valuable support for our study. We have incorporated this reference into the discussion section to further explain the increased distance observed between personal belongings. Specifically, we added the following paragraph as in the "Revised version".

**Revised Version:**

 Several studies have confirmed the increase in personal space during the COVID-19 pandemic and suggested the practice of social distancing attributed to the increase (Fini, Tummolini, and Borghi, 2021; Fazio et al., 2021). Moreover, our study found that the distance between objects increased during the pandemic. **Recent findings from studies on the psychological impact of the COVID-19 pandemic provide additional support for our findings. For example, Serino et al. (2021) demonstrated that the pandemic led to a sharpening of peripersonal space boundaries, with individuals adapting their spatial perceptions in response to increased health-related concerns. This phenomenon aligns with our observation that the distance between personal belongings increased during the pandemic. The findings suggest that participants might have subconsciously extended their spatial boundaries to maintain greater safety and reduce perceived risks associated with potential contamination. This adaptation, as highlighted by Serino et al. (2021), may explain why people positioned their belongings farther away from other's during the pandemic.** Considering Ariga (2016), personal belongings have the same personal space-like properties as the owner, belongings also maintain their social distance when they are placed. This distance could manifest in the elongated distance of personal belongings.

 5. The fact that participants wore face masks should be mentioned earlier in the text, specifically in the Methods section, as it is a relevant procedural detail. Additionally, this should be discussed further in the Discussion section, with reference to studies examining the relationship between mask-wearing and interpersonal or peripersonal distance.

**Response:**

 We revised the Methods section to include this procedural detail. Additionally, we expanded the Discussion section to explore the potential influence of mask-wearing on interpersonal and peripersonal distance, supported by Cartaud et al.(2020).

**Revised version:**

**Methods**

 The inclusion criteria were native Japanese or international students who could speak Japanese proficiently. They were paid 1000 yen to agree to participate. **All participants were required to wear face masks throughout the experiment as part of standard COVID-19 precautions.**

**Discussion**

 In fact, all valid participants in this study voluntarily wore face masks, which is a routine infection control measure. **Beyond compliance, mask-wearing might have also influenced the observed spatial distancing behavior. Research has shown that wearing face masks can alter interpersonal perceptions and decrease interpersonal and peripersonal distances in certain contexts. For example, Cartaud et al. (2020) found that individuals perceived as wearing face masks are considered more trustworthy, leading to reduced physical distance. Similarly, Biggio et al. (2022) reported that mask-wearing shapes interpersonal space by fostering a sense of safety and reducing the need for extended spatial boundaries. In our study, while participants wore face masks during the experiment, the observed increase in distance between personal belongings suggests that compliance with infection control measures and risk-avoidance behaviors might interact differently when participants place objects instead of interacting directly with people.**

 Minor:

 6.I found the observations about the role of smartphones and their potential impact on personal space particularly insightful in the discussion.

**Response:**

 Thank you for your feedback. Our manuscript mentions that smartphones and social media may affect interpersonal distance, but not in conjunction with the concept of interpersonal/intimate distance. To that end, we modified and expanded the section (the modifications here partly same as the response to the question 1, but we're still showing it to you here) :

**Revised Version:**

**Furthermore, the perception of distance may be shaped by long-term societal and technological changes. The widespread use of smartphones and social media in the 2010s might have altered norms around physical proximity and social interaction. Future research could investigate whether personal space norms adapt to such technological shifts and how these changes interact with interpersonal and peripersonal distances.**

 7. I suggest reconsidering the inclusion of Figure 3, as it primarily displays the results of a manipulation check, and may not add value to the main narrative of the paper.

**Reponse:**

 Thank you for your comments. Figure 3 includes part of the results described in the text but also provides a visualization of the data distribution for the manipulation check. To streamline the manuscript, we removed Figure 3 while retaining the textual description, as we believe that the results of the manipulation check are crucial for demonstrating the validity of our experiment design.

**Revised version:**

 …we calculated the mean favorability for each condition (friendly and unfriendly).

# Reviewer report: Reviewer response for version 1

**DOI:** 10.5256/f1000research.143432.r259293

**Published:** 2024-05-14

**Authors:** Tomoko Isomura

**Affiliations:** 1 Graduate School of Informatics, Nagoya University, Nagoya, Japan

Summary:

 This pre-registered study aimed to examine the effect of the COVID-19 pandemic on human behavior, specifically how long people prefer to keep their personal belongings distanced from others. The authors set up a scenario in which participants placed their personal belongings on a desk after a confederate had done the same, without being aware of the study's purpose. They measured the physical distance between the belongings of the participant and the confederate. The obtained data were compared with those from a previous study (Ariga, 2016), conducted before the pandemic using identical procedures. The results showed that the distance between belongings in the present study was greater than reported in the previous study. The authors concluded that the pandemic has increased the personal space people maintain, which further extends the distance they keep from others' belongings. Additionally, although the authors manipulated the confederate’s friendliness—a factor that the previous study demonstrated had an effect—this manipulation did not induce any differences in the present study.

 I found this study interesting, particularly in that the methods they employed were well-designed to capture human’s implicit attitude for the boundaries of self and others. The manuscript is generally well written, and it is valuable that the authors pre-registered this study. However, I have some concerns about this manuscript in its current form, as follows.

 Firstly, I question whether the terms "extended" or "extension of" personal space accurately represent the phenomenon described. I acknowledge that our representation of the bodily self can be modulated when manipulating objects or interacting with others, potentially resulting in an "extension," enlargement, or prolongation—or even the opposite—of one's peri-personal or personal space. However, the mental attitude of keeping one's belongings separate from others', especially when the objects are placed away from the body (e.g., on a desk when temporarily leaving), does not seem to me to constitute an "extension" of personal space, although I agree that it reflects some processing of self-other boundaries. It would be advantageous if the authors could include additional literature to support this concept, beyond Ariga (2016). On a related but more minor point, the authors state in the second paragraph of the Introduction that Sasaki et al., 2018 demonstrated that a sense of body ownership is conferred to objects. However, I believe that this study is more appropriately interpreted as showing that the sense of "object ownership" is driven by body-related processing, and the authors' interpretation may be somewhat misleading.

 Secondly, and more importantly, I found it highly challenging to assert that the effect observed by the authors is truly driven by the COVID-19 pandemic, given the limited measures they collected. While I acknowledge the inherent challenges in proving such causation, I suggest that the authors provide more information and/or conduct additional analyses to make the study more persuasive. Firstly, the authors should clearly state the size of the table and the position of the chair, ensuring they are indeed identical to those used in Ariga (2016). The authors should include this information in the main text, not just within a figure. I assume that the relative position of the chairs to the table would affect the participant’s behavior, as people tend to place objects directly in front of where they are seated. Thus, it is essential that these parameters were controlled between the studies. I also suggest the authors provide more information about the confederates, including their sexes and ages, because Ariga (2016) demonstrated that the relationship between the confederates' and participants' sexes had a clear effect on the results. Furthermore, I speculate that the size or number of the participants' belongings could affect the distance measures. It seems that Ariga (2016) considered only the participant’s bag, but what was the case in this study? If the authors have pictures of the placement of the belongings, they might be able to conduct additional analyses that could potentially rule out the influence of the size/number of the participant’s belongings on the findings.

 Ideally, it would have been valuable if the authors had collected some subjective scales related to the pandemic, such as how afraid the participants were of or how cautious they were about the infection. This could be a subject to consider for future studies.

 Minor points:

1. In the third paragraph of the Introduction, the authors reference Tootell et al., 2021, as a study that reported an increase in interpersonal distance during the pandemic. However, I believe that this study was conducted before the pandemic. Could it be that the authors intended to cite Holt et al., 2022 instead?
2. The authors should clearly specify the timing of data collection within the Methods section of the main text.
3. Upon reviewing the raw data provided by the authors, I noticed that it includes 70 data. If I understood correctly, there should only be 68 data since 2 participants did not bring any belongings. Please check this.
4. This study did not find any effect of the confederate's friendliness on the participants' behavior. The authors claims that this result is inconsistent with findings from Ariga (2006), potentially due to a ceiling effect. However, since the ANOVA revealed only a main effect of experimental timing and the interaction was not significant, it may be misleading to definitively state that the findings are inconsistent regarding the effect of the confederate's friendliness.

Is the work clearly and accurately presented and does it cite the current literature?

Yes

If applicable, is the statistical analysis and its interpretation appropriate?

Partly

Are all the source data underlying the results available to ensure full reproducibility?

Partly

Is the study design appropriate and is the work technically sound?

Partly

Are the conclusions drawn adequately supported by the results?

Partly

Are sufficient details of methods and analysis provided to allow replication by others?

Partly

Reviewer Expertise:

experimental psychology; embodied cognition; social cognition

I confirm that I have read this submission and believe that I have an appropriate level of expertise to confirm that it is of an acceptable scientific standard, however I have significant reservations, as outlined above.

# Response

**Published:** 2024-08-12

**Authors:** Kaito Takashima

**Affiliations:** Kyushu University, Japan

**Replies to the comments by Dr.Tomoko Isomura (Graduate School of Informatics, Nagoya University, Nagoya, Japan):**

**Reviewer Comments:** 

**“This pre-registered study aimed to examine the effect of the COVID-19 pandemic on human behavior, specifically how long people prefer to keep their personal belongings distanced from others. The authors set up a scenario in which participants placed their personal belongings on a desk after a confederate had done the same, without being aware of the study's purpose. They measured the physical distance between the belongings of the participant and the confederate. The obtained data were compared with those from a previous study (Ariga, 2016), conducted before the pandemic using identical procedures. The results showed that the distance between belongings in the present study was greater than reported in the previous study. The authors concluded that the pandemic has increased the personal space people maintain, which further extends the distance they keep from others' belongings. Additionally, although the authors manipulated the confederate’s friendliness—a factor that the previous study demonstrated had an effect—this manipulation did not induce any differences in the present study.**

**I found this study interesting, particularly in that the methods they employed were well-designed to capture human’s implicit attitude for the boundaries of self and others. The manuscript is generally well written, and it is valuable that the authors pre-registered this study. However, I have some concerns about this manuscript in its current form, as follows.”**

**“Firstly, I question whether the terms "extended" or "extension of" personal space accurately represent the phenomenon described. I acknowledge that our representation of the bodily self can be modulated when manipulating objects or interacting with others, potentially resulting in an "extension," enlargement, or prolongation—or even the opposite—of one's peri-personal or personal space. However, the mental attitude of keeping one's belongings separate from others', especially when the objects are placed away from the body (e.g., on a desk when temporarily leaving), does not seem to me to constitute an "extension" of personal space, although I agree that it reflects some processing of self-other boundaries. It would be advantageous if the authors could include additional literature to support this concept, beyond Ariga (2016). On a related but more minor point, the authors state in the second paragraph of the Introduction that Sasaki et al., 2018 demonstrated that a sense of body ownership is conferred to objects. However, I believe that this study is more appropriately interpreted as showing that the sense of "object ownership" is driven by body-related processing, and the authors' interpretation may be somewhat misleading.”**

**Author Reply:**

 Thank you very much for your insightful comments.

 Before starting this study, we extensively debated the differences between the sense of ownership and personal space and territory, ultimately concluding that this phenomenon is related to personal space. Here, we outline our rationale: The most significant evidence that the extended space is part of personal space is its variability depending on the level of familiarity with others. The size of personal space is not fixed; it varies depending on the level of familiarity with others. Ariga (2016) reported that this feature is also reflected in owned objects. Our focus on this feature is why we use the term "personal space."

 While it is true that the "extension of personal space" shares similar characteristics with personal space in terms of variability based on familiarity with others, we agree with your point that the "extension of personal space" discussed in our study is not entirely the same as "personal space."

 As you pointed out, concepts such as the sense of ownership and territory are likely relevant to this phenomenon. To prevent readers from misunderstanding the same confusion as you, we have clarified the concept of “extension of personal space” in the original article:

 The end of the second paragraph in the introduction: “…extended to the space surrounding their belongings (i.e., extended personal space). The placement of such personal belongings can be considered the most significant evidence of an extension of personal space, as it shares the same characteristics with personal space (Russell & Ward, 1982; Becker, 1973), that the variability depending on the level of familiarity with others.”

 Additional references:

 Russell, J. A., & Ward, L. M. (1982). Environmental psychology. Annual Review of Psychology, 33, 651–688. https://doi.org/10.1146/annurev.ps.33.020182.003251

 Becker, F. D. (1973). Study of spatial markers. Journal of Personality and Social Psychology, 26, 439–445.

 As for the second point you mentioned about the citation of Sasaki et al (2018), after checking, we agree that this citation is not optimal and potentially misleading here. The argument for this statement is also mentioned later in the paragraph, so we have deleted this sentence.

**Reviewer Comments:** 

**“Secondly, and more importantly, I found it highly challenging to assert that the effect observed by the authors is truly driven by the COVID-19 pandemic, given the limited measures they collected. While I acknowledge the inherent challenges in proving such causation, I suggest that the authors provide more information and/or conduct additional analyses to make the study more persuasive. Firstly, the authors should clearly state the size of the table and the position of the chair, ensuring they are indeed identical to those used in Ariga (2016). The authors should include this information in the main text, not just within a figure. I assume that the relative position of the chairs to the table would affect the participant’s behavior, as people tend to place objects directly in front of where they are seated. Thus, it is essential that these parameters were controlled between the studies. I also suggest the authors provide more information about the confederates, including their sexes and ages, because Ariga (2016) demonstrated that the relationship between the confederates' and participants' sexes had a clear effect on the results.”**

**Author Reply:**

 Thank you very much for pointing out the inadequacies in our description. For the purpose of controlling the possible influence of the experimental setting on the results, in the present experiment, we used a table of exactly the same size as Ariga (2016), and the positions of the chairs and table were exactly the same as in Ariga's (2016) study. Indeed, including these contents in the main text can help readers better eliminate concerns about the impact of experimental settings on the results, so we clearly stated the contents of this part in the main text. That said, we should consider other factors, than COVID-19, like mood, anxiety, or other person-related characteristics.

 Moreover, in our experiment, the confederates were three graduate male students who took turns according to their schedules. Their average age was 23.6 years old (the confederate in the original experiment was a 21-year-old male). We also added information about the gender and age of the confederates in the original text.

**Reviewer Comments:** 

**“Furthermore, I speculate that the size or number of the participants' belongings could affect the distance measures. It seems that Ariga (2016) considered only the participant’s bag, but what was the case in this study? If the authors have pictures of the placement of the belongings, they might be able to conduct additional analyses that could potentially rule out the influence of the size/number of the participant’s belongings on the findings.”**

**Author Reply:**

 Although we asked participants to come with their bags, we did not force them. Participants came with their bags, jackets, notebooks, and so on. Because we did not record the type of belongings as well as the size of them, it is difficult to analyze the effects of them. Instead, to control the effect of the size of the items on the results, we standardized the measurements by only measuring the minimum distance between two objects and did not include the size of the objects themselves. We believe that with this approach we can minimize the effect of object size on the results and make full use of the sample size.

**Reviewer Comments:**

**“Ideally, it would have been valuable if the authors had collected some subjective scales related to the pandemic, such as how afraid the participants were of or how cautious they were about the infection. This could be a subject to consider for future studies.”**

**Author Reply:**

 Unfortunately, we did not measure subjective scales related to the pandemic. We added this point in the Discussion section for future research. Instead, the trend of the pandemic was added in the main text, following the other reviewer.

**Reviewer Comments:**

***Minor points:***

**“In the third paragraph of the Introduction, the authors reference Tootell et al., 2021, as a study that reported an increase in interpersonal distance during the pandemic. However, I believe that this study was conducted before the pandemic. Could it be that the authors intended to cite Holt et al., 2022 instead?”**

**Author Reply:**

 Thank you very much for pointing out our misunderstanding. Although this article (Tootell et al., 2021) was conducted during the period of COVID-19, the conclusion does not indicate the relationship between the increased interpersonal distance and COVID-19. Therefore, we accept your kind suggestion to cite Holt et al. (2022) and add a reference to Lachini et al. (2021) to increase the persuasiveness of the previous study. The details of the references are as follows:

 Iachini, T., Frassinetti, F., Ruotolo, F., Sbordone, F. L., Ferrara, A., Arioli, M., ... & Ruggiero, G. (2021). Social distance during the COVID-19 pandemic reflects perceived rather than actual risk. *International Journal of Environmental Research and Public Health, 18*(11), 5504.

 Holt, D. J., Zapetis, S. L., Babadi, B., Zimmerman, J., & Tootell, R. B. (2022). Personal space increases during the COVID-19 pandemic in response to real and virtual humans. *Frontiers in Psychology, 13*, 952998.

 We also revised our manuscript.

**Reviewer Comments:**

**“The authors should clearly specify the timing of data collection within the Methods section of the main text.”**

**Author Reply:**

 Thank you for your advice. Although the experiment period was referred to in the abstract, we added the timeline of data collection in the method- participants part of the main text as follows: The experiment period was from June 10, 2022, to January 23, 2023.

**Reviewer Comments:**

**“Upon reviewing the raw data provided by the authors, I noticed that it includes 70 data. If I understood correctly, there should only be 68 data since 2 participants did not bring any belongings. Please check this.”**

**Author Reply:**

 What you understand is correct. Two of the participants did not take any of their belongings to take part in the experiment. Therefore, the experimental conditions were not met, and we excluded the data from these two participants. Moreover, we have mentioned the exclusion in the main text as follows: we collected data from 70 participants; of these, two participants were excluded since they did not bring any belongings.

**Reviewer Comments:**

**“This study did not find any effect of the confederate's friendliness on the participants' behavior. The authors claims that this result is inconsistent with findings from Ariga (2006), potentially due to a ceiling effect. However, since the ANOVA revealed only a main effect of experimental timing and the interaction was not significant, it may be misleading to definitively state that the findings are inconsistent regarding the effect of the confederate's friendliness.”**

**Author Reply:**

 Our study indeed did not find a significant effect of the confederate's friendliness on the participants' behavior, as indicated by the lack of a significant interaction in the ANOVA. We acknowledged this finding and compared it with the results from Ariga (2006), where friendliness did have an effect. In our discussion, we suggested that the inconsistency with Ariga (2006) might be due to a ceiling effect. The ceiling effect suggests that participants may have already reached a maximum level of performance or behavior, making it difficult to observe any additional effect of the confederate's friendliness. While this is a plausible explanation, we agree that it should be presented as a hypothesis rather than a definitive conclusion.

 To address your concern about the conclusiveness of our statement, we propose revising our discussion to indicate that the findings are suggestive rather than conclusive. Here is the revised statement: “Intriguingly, our study did not find a significant effect of the confederate's friendliness on participants' behavior, as indicated by the lack of a significant interaction effect in the ANOVA. This result appears to be inconsistent with the findings from Ariga (2016). One possible explanation for this discrepancy could be a ceiling effect, where participants' behavior might have already reached its peak, limiting the observable impact of additional friendliness.”

# Reviewer report: Reviewer response for version 1

**DOI:** 10.5256/f1000research.143432.r186686

**Published:** 2023-07-24

**Authors:** Leon O. H Kroczek

**Affiliations:** 1 University of Regensburg, Regensburg, Germany

**Summary: **In the present, pre-registered study, participants were asked to place their belongings on a table together with the belongings of a confederate while the physical distance between personal belongings is measured as an outcome variable. These data are then compared to pre-pandemic data of the same paradigm from Ariga (2016). The authors find an increase in physical distance in the current compared to the original study and interpret these findings in the light of changes of social distances induced by the Covid-19 pandemic. The authors also investigate a within-subject manipulation, i.e. the influence of confederate friendliness on distance between belongings but do not find a significant effect and thus cannot replicate the results of Ariga (2016).

**Review:**

 I think the present study uses an interesting and unobtrusive way to study interpersonal distance. I also applaud the authors for pre-registering their study. However, one major limitation of this study is that the main comparison is with respect to an unmatched group of participants almost 10 years ago, at a different university. The effects *could* be driven by changes in social distancing introduced by the Covid-19 pandemic, but there are also several other effects that could influence the data. The authors report that the sample had the same sex ratio as the original study, but what about other factors like mood, (social) anxiety, age, other person-related characteristics? Also trivial points, like whether the same kind of belongings were used between studies (jackets or handbags) might have influenced the results (e.g. jackets might fall from the table when placed too close to the border). Some of this is already discussed in the limitations but needs to be highlighted when making claims about Covid-19 related changes of behavior.

 In my view, it would be much more convincing if the authors could show that distance is influenced by a subjective variable related to perceived vulnerability to Covid-19 (e.g. individual fear of an infection, belief about importance of social distancing as a policy against spread of infection, fear for family members,…, but also belief about whether belonging can be the source of infections) or an objective variable like cases reported in the news, number of active restrictions due to the pandemic, availability of vaccines. It seems also to be the case that standard deviations were bigger in the present study, suggesting a stronger influence of individual characteristics. Anyway, this point needs to be made more clearly throughout the manuscript and should even be reported in the abstract. Side note: Looking in the pre-registration the effect of the within-subject factor “alcohol disinfection” could be also informative about that.

 As a related point, it would be great to get more information on the state of the pandemic at the test center during the test period. How many new infections were reported during this time? What were the active policies against spread of infections, was the topic very present in the media? I ask this because in Germany, in the second half of 2022 the pandemic was less of a topic than in the years before and there were (almost) no impacts of Covid-19 related restriction in everyday life. I wonder whether similar claims could be made about Japan during this time?

 In addition, it would strengthen the argument of the authors to describe how the actual distance between persons was related to the distance between belongings. Was the distance in belonging correlated to actual distancing? Please also include more details on the paradigm here: How big was the distance between chairs in the experimental setting? Was it closer or further than a minimum recommended distance? Could this influence the placing of belongings?

 The authors suggest that a ceiling effect precluded them from finding an effect of friendliness as in Ariga 2016. Looking at the distributions most of the distances were below 60 cm with the table being 150 cm wide (but please state where exactly on the table the belongings of the confederate where located), and original effect being around 10 cm. These data seem to suggest that the effect would have been observable if present. Another interpretation might therefore be that the belongings may not reflect personal space after all (contrary to Ariga, 2016). This should be discussed.

 Minor:

- I suggest to change the wording “peacetime” to “pre-pandemic” throughout
- I had difficulties understanding the first paragraph, maybe consider rephrasing it. Also there is more recent evidence on the relation of peripersonal space and interpersonal distance  (e.g. see works of Yann Coello)

Is the work clearly and accurately presented and does it cite the current literature?

Partly

If applicable, is the statistical analysis and its interpretation appropriate?

Yes

Are all the source data underlying the results available to ensure full reproducibility?

Yes

Is the study design appropriate and is the work technically sound?

No

Are the conclusions drawn adequately supported by the results?

No

Are sufficient details of methods and analysis provided to allow replication by others?

Partly

Reviewer Expertise:

experimental psychology, social interaction research

I confirm that I have read this submission and believe that I have an appropriate level of expertise to state that I do not consider it to be of an acceptable scientific standard, for reasons outlined above.

# Response

**Published:** 2024-08-12

**Authors:** Kaito Takashima

**Affiliations:** Kyushu University, Japan

**Replies to the comments by Dr. Leon O. H Kroczek (University of Regensburg, Regensburg, Germany):**

**Reviewer Comment:**

“I think the present study uses an interesting and unobtrusive way to study interpersonal distance. I also applaud the authors for pre-registering their study. However, one major limitation of this study is that the main comparison is with respect to an unmatched group of participants almost 10 years ago, at a different university. The effects *could* be driven by changes in social distancing introduced by the Covid-19 pandemic, but there are also several other effects that could influence the data. The authors report that the sample had the same sex ratio as the original study, but what about other factors like mood, (social) anxiety, age, other person-related characteristics? Also trivial points, like whether the same kind of belongings were used between studies (jackets or handbags) might have influenced the results (e.g. jackets might fall from the table when placed too close to the border). Some of this is already discussed in the limitations but needs to be highlighted when making claims about Covid-19 related changes of behavior.”

**Author Reply:**

 Thank you for your comments. Unfortunately, the current evidence cannot assess the possibility that differences in data collection year, person-related characteristics, and university might influence the distance between inter-belongings. In addition, although the participants’ belongings were limited to bags in the previous study (Ariga, 2016), in the present study, we did not restrict participants to carry only bags. We agree with your comment that it is important to highlight this information when making claims about COVID-19-related changes in behavior. Thus, in the conclusion part, we added, “…the results show that people significantly increased, with a large effect size, the distance between personal belongings during the COVID-19 pandemic. It is important to note, however, that the results of the present experiment do not fully demonstrate that COVID-19 was the only factor that affected the expanded personal space between belongings.”

 As we stated in the Discussion section, our hypothesis was that the distance between objects to which the interpersonal relationship expands increased during the COVID-19 period, but we considered COVID-19 as just one possible reason for this. To avoid misunderstanding among readers and other researchers, we adjusted the tone of the descriptions related to the plausible explanations of our results in the Discussion section of the revised manuscript:" ... extended personal space increased during the period of COVID-19 pandemic, but we consider COVID-19 as one of the reasons for this change. "

**Reviewer Comment:**

**“In my view, it would be much more convincing if the authors could show that distance is influenced by a subjective variable related to perceived vulnerability to Covid-19 (e.g. individual fear of an infection, belief about importance of social distancing as a policy against spread of infection, fear for family members,…, but also belief about whether belonging can be the source of infections) or an objective variable like cases reported in the news, number of active restrictions due to the pandemic, availability of vaccines. It seems also to be the case that standard deviations were bigger in the present study, suggesting a stronger influence of individual characteristics. Anyway, this point needs to be made more clearly throughout the manuscript and should even be reported in the abstract. Side note: Looking in the pre-registration the effect of the within-subject factor “alcohol disinfection” could be also informative about that.”**

**Author Reply:**

 Several studies related to personal distance during COVID-19, such as Fini et al. (2021), have shown that interpersonal distance is influenced by the perception of Realistic Threat, as measured through the COVID-19 threat. Moreover, previous research suggests that the public's practice of social distancing is not solely driven by the perception of infection but also by the social meaning of collective health (Fazio et al., 2021).

 We have added the following to the third paragraph of the introduction:

 “... which means that exposed people and objects may carry the coronavirus present in aerosols. Doremalen et al. (2020) compared the survivability of SARS-CoV-2 (leading to COVID-19) and SARS-CoV-1 on different surfaces. They found that the survival time of SARS-CoV-2 on various material surfaces ranged from a few hours to a few days, suggesting that the surface of an item can be a potential pathway for virus transmission. Centers for Disease Control and Prevention (CDC) and the World Health Organization (WHO) have also mentioned that the virus can be transmitted through direct contact with contaminated surfaces.”

 We greatly appreciate your suggestion to include these previous research findings in the manuscript and the abstract. This will help clarify to readers that the increased distance between belongings during COVID-19 may be influenced by the perceived threat that objects can also be a source of infection.

 Regarding the “alcohol disinfection” condition, it belonged to another experiment in our lab. For this experiment, we can only provide the results, which indicate that expanded personal space does not change depending on hand disinfection.

**Reviewer Comment:**

**“As a related point, it would be great to get more information on the state of the pandemic at the test center during the test period. How many new infections were reported during this time? What were the active policies against spread of infections, was the topic very present in the media? I ask this because in Germany, in the second half of 2022 the pandemic was less of a topic than in the years before and there were (almost) no impacts of Covid-19 related restriction in everyday life. I wonder whether similar claims could be made about Japan during this time?”**

**Author Reply:**

 We utilized Google Trends to track the frequency of the keyword "コロナ" (Japanese term for COVID-19) in Google searches. The data revealed that media coverage of COVID-19 in Japan fluctuated significantly, with the peak occurring at the onset of the pandemic in early 2020. Our experiment took place from June 2022 to January 2023, during which time the final wave of COVID-19 peaked, and media coverage remained high. However, by January 2023, media coverage started to decrease. Additionally, COVID-19-related information was widely disseminated not only through news and entertainment media but also in public places such as restaurants and shopping malls, where social distancing and health and safety measures were prominently displayed. According to Japanese news reports ***[Please refer to the below links for Image 1 and Image 2]***, the number of new COVID-19 infections across Japan exceeded 40,000 per day starting in June 2022, reaching over 100,000 per day by July 2022. This marked the highest number of cases since the initial outbreak of COVID-19 in Japan in 2020.

 Image 1 Reference link: https://f1000research.s3.amazonaws.com/linked/669428.Image_1.pdf 

 Image 2 Reference link: https://f1000research.s3.amazonaws.com/linked/669429.Image_2.pdf 

**Reviewer Comment:**

**“In addition, it would strengthen the argument of the authors to describe how the actual distance between persons was related to the distance between belongings. Was the distance in belonging correlated to actual distancing?”**

**Author Reply:**

 While we believe that our current findings reflect the physical distance between the confederate and the participant, we did not measure this distance during the actual experiment. Instead, we fixed their physical distance to eliminate any potential influence that physical distance might have had on the placement of objects, as it could have affected the results.

**Reviewer Comment:**

**“Please also include more details on the paradigm here: How big was the distance between chairs in the experimental setting? Was it closer or further than a minimum recommended distance? Could this influence the placing of belongings?”**

**Author Reply:**

 The distance between the two chairs was approximately 30-40 cm, significantly less than the recommended social distancing measure of 2 meters due to the constraints of the experiment room's size and layout. The small distance between chairs may have created a sense of social anxiety and constriction at the beginning, making participants place belongings farther away to alleviate seated proximity in subsequent place sessions. No studies have been done to illustrate the effect of physical proximity on the unraveling of the distance between items. We added this part of the influence of chair distance on the distance of placing objects in the limitation part.

 "... as they were unsure whether the stranger and their belongings were infected with viruses. Moreover, some previous studies declared that the confined spaces may enhance the feeling of personal space invasion, thereby increasing psychological stress and anxiety (Aiello et al ., 1977; Evans, & Wener, 2007). In the present study, for strict replication and fair comparison, the chair distance was set to be the same as in the original experiment conducted in the pre-pandemic period, but the small distance between chairs may have created a sense of social anxiety and constriction at the beginning, making participants place belongings farther away to alleviate seated proximity in subsequent place sessions. No studies that have been done to illustrate the effect of physical proximity on the unraveling of the distance between items.

 "... and sat next to the confederate’s chair. The distance between two chairs was roughly 30-40 cm." in the Procedure section.

**Reviewer Comment:**

**“The authors suggest that a ceiling effect precluded them from finding an effect of friendliness as in Ariga 2016. Looking at the distributions most of the distances were below 60 cm with the table being 150 cm wide (but please state where exactly on the table the belongings of the confederate where located), and original effect being around 10 cm. These data seem to suggest that the effect would have been observable if present. Another interpretation might therefore be that the belongings may not reflect personal space after all (contrary to Ariga, 2016). This should be discussed.”**

**Author Reply:**

 Thank you for your insightful comment. Our manuscript lacked a crucial piece of information: the size of the confederate's belongings, which is approximately 36 cm wide. We have now included this detail in our manuscript. Although the confederate experimenters tried to place their belongings as far as possible to the left corner of the desks, the space left for the participants was estimated to be around 100-110 cm. Additionally, the maximum distance between the belongings would be approximately 60 cm, assuming the participant's belongings were about 40 cm wide. Attempting to increase the distance by 10 cm from the unfriendly confederate's belongings to the average brings it close to the maximum possible distance. Participants might have avoided placing their belongings in the corners due to concerns about them falling. We believe that if the desks had been longer, participants could have placed their belongings further from the unfriendly confederate's belongings without the risk of them falling. Based on this context, we interpreted the results as being influenced by a ceiling effect. Your comment helped us identify and address this missing information.

**Reviewer Comment:**

**Minor:**

**“I** **suggest to change the wording “peacetime” to “pre-pandemic” throughout.”**

**Author Reply:**

 We changed it to “pre-pandemic”.

**Reviewer Comment:**

**“I** **had difficulties understanding the first paragraph, maybe consider rephrasing** **it. Also there is more recent evidence on the relation of peripersonal space** **and interpersonal distance (e.g. see works of Yann Coello)”**

**Author Reply:**

 Thank you for your comments. We have rewritten the first paragraph and added new studies to help clarify our logic for you and the readers.

 “Social psychology and proxemics have long addressed personal space as the spatial range surrounding the individual (Sommer, 1959; Hall, 1966). The space around them represents a mediation zone between the body and the environment, contributing to the organization of social life. For example, when this space is encroached by others, we may feel uncomfortable or may prepare our bodies for defensive responses (Hall, 1966; Coello and Cartaud, 2021). However, the center of personal space is oneself, but it is not restricted to the physical body; much evidence proposed that the concept of self is expanding in space and timeline. For example, the ‘cutaneous rabbit’ can be felt not only on one’s body but also on an object that one is grasping (Miyazaki et al., 2010). The line of sight away from the eye can still be perceived as exerting force on the object (Guterstam et al., 2019). Furthermore, the extension of body representations to objects has been observed in neuroscience (Iriki et al., 1996). As a result, personal space boundaries also expand with the extension of body perception.”
